# Update of inflammasome activation in microglia/macrophage in aging and aging‐related disease

**DOI:** 10.1111/cns.13262

**Published:** 2019-11-14

**Authors:** Meng‐yan Hu, Yin‐yao Lin, Bing‐jun Zhang, Dan‐li Lu, Zheng‐qi Lu, Wei Cai

**Affiliations:** ^1^ Department of Neurology Center for Mental and Neurological Disorders and Diseases The Third Affiliated Hospital of Sun Yat‐sen University Guangzhou China; ^2^ Center of Clinical Immunology Center for Mental and Neurological Disorders and Diseases The Third Affiliated Hospital of Sun Yat‐sen University Guangzhou China

**Keywords:** aging, inflammasome, inflammation, macrophage, microglia

## Abstract

Aging and aging‐related CNS diseases are associated with inflammatory status. As an efficient amplifier of immune responses, inflammasome is activated and played detrimental role in aging and aging‐related CNS diseases. Macrophage and microglia display robust inflammasome activation in infectious and sterile inflammation. This review discussed the impact of inflammasome activation in microglia/macrophage on senescence “inflammaging” and aging‐related CNS diseases. The preventive or therapeutic effects of targeting inflammasome on retarding aging process or tackling aging‐related diseases are also discussed.

## 1 INTRODUCTION

The aging process of central nervous system (CNS), as well as the pathology of aging‐related diseases, is closely associated with inflammatory responses.^1^, ^2^ Inflammasome is a multiprotein complex which is induced in response to microbial invasion or damage‐associated molecular patterns (DAMPs) in innate immune cells.^3^ Activation of inflammasome results in production of proinflammatory factors, including interleukin (IL)‐1β, and IL‐18, which represents an important amplifier of inflammation. Notably, inflammasome is activated during aging and aging‐related CNS diseases, accelerating the process of senility and CNS disorders at the same time. A broad body of studies have confirmed the key role of microglia and macrophage in aging and aging‐related diseases. Inflammatory milieu during aging‐related CNS diseases activates microglia macrophage, while activation of microglia and macrophage contributes to the exacerbation of neural inflammation in aging‐related disease.^4^, ^5^, ^6^ Microglia and macrophage are the main cells in which inflammasome is potently activated. This review summarizes the impact of inflammasome activation in microglia/macrophage during aging and aging‐related disorders. Preventive or therapeutic effects of targeting inflammasome on tackling aging‐related diseases are also discussed.

## 2 INFLAMMASOME IS AN AMPLIFIER OF NEURAL INFLAMMATION

Inflammasome is an intracellular complex that detects physiological and pathogenic stimuli. Inflammasome activation was first discovered in myeloid cells, including macrophage/microglia, neutrophil, and dendritic cell.^7^ Recently, it was demonstrated that other cell types, including, oligodendrocyte, astrocyte, neurons, and epithelial cell, could also trigger inflammasome activation.^8^, ^9^, ^10^, ^11^ Among inflammasome‐forming cells, it is microglia/macrophage that has the most potent inflammasome activation, thus is most widely studied.^12^

Classically, inflammasome is composed of sensor, executor, and substrate. Multiple sensors have been found to detect stimuli for inflammasome, including NACHT, LRR, and PYD domains‐containing protein 1 (NLRP1), NLRP2, NLRP3 NLR family CARD domain‐containing protein 4 (NLRC4), and absent in melanoma 2 (AIM2).^13^ Canonically, sensor of inflammasome recruits the executive enzyme of Caspase‐1 with the adaptor of apoptosis‐associated speck‐like protein, also known as PYCARD (ASC). Subsequently, Caspase‐1 cleaves the substrates of pro‐IL‐1β and pro‐IL‐18 into their active form (Cleaved‐IL1β and Cleaved‐IL18). It is found that Caspase‐8 and Caspase‐11 could also participate in the process of inflammasome activation as executors. Moreover, gasdermin‐D (GSDMD) could be activated by the caspase enzymes (eg, Caspase‐11) and formed pores in cytomembrane of inflammasome‐activating cells, resulting in specific cell death process called pyroptosis.^14^ Other accessories of inflammasome have been discovered. NIMA‐related kinase 7 (NEK7) has been found to bridge adjacent NLRP3 for their oligomerization and mediate subsequent inflammasome activation.^15^, ^16^

**Figure 1:**
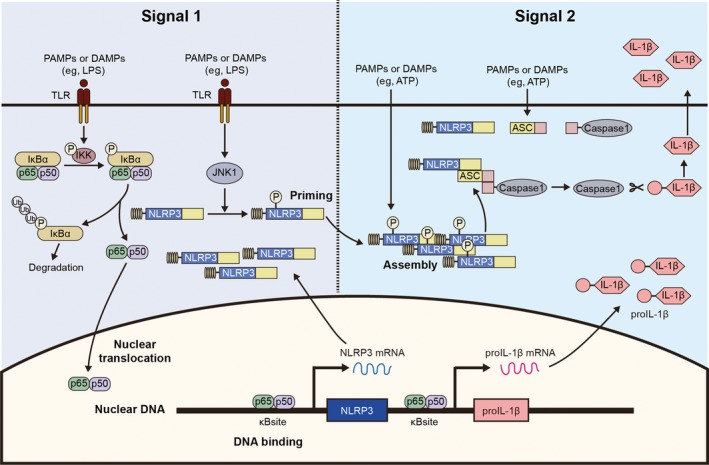
Two‐signal model of inflammasome signaling. The classic understanding of inflammasome is based on a two‐signal model. In signal 1, sensors (eg, NLRP3) of inflammasome are activated by PAMPs/ DAMPs, leading to activation of NF‐κB pathway and increased transcription of inflammasome‐relevant genes such as NLRP3 and pro‐IL1β. In signal 2, PAMPs/DAMPs (eg, ATP) further activate the inflammasome sensors. The sensors then undergo oligomerization and attach to ASC. ASC acts as a molecular platform that recruits pro‐caspase enzymes. The pro‐caspase enzymes are then cleaved into their active form which subsequently cleave pro‐IL1β and pro‐IL18 into cleaved‐IL1β and cleaved‐IL18

The classic understanding of the process of inflammasome formation is based on a two‐signal model (Figure 1). In signal 1, sensors of inflammasome are activated by pathogen‐associated molecular patterns (PAMPs), the signal is passed through by NF‐κB pathway, and transcription of inflammasome‐relevant genes such as NLRP3 and pro‐IL1β is increased.^16^, ^17^ In signal 2, DAMPs (including ATP, ROS, Ca^2+^ mobilization, uric acid, alums, and silica)^18^ further activate the inflammasome sensors. The sensors then undergo oligomerization and attach to ASC. ASC acts as a molecular platform that recruits pro‐caspase enzymes. The pro‐caspase enzymes are then cleaved into their active form which subsequently cleaves pro‐IL1β and pro‐IL18 into cleaved‐IL1β and cleaved‐IL18. The inflammasome products further exert their inflammatory amplifying effects. In the real battlefield of disease/injury, it is more likely for cells to come across the two kinds of signal concurrently, and the two signals are transmitted at the same time. Moreover, noncanonical activating process of inflammasome is discovered. Lipid A could activate Caspase‐4/5/11 directly, inducing oligomerization of the caspase enzymes, which activates cysteine protease to cleave the downstream substrate of GSDMD.^19^, ^20^ Therefore, the two‐signal theory is questioned. Nevertheless, the two‐signal theory of inflammasome activation stills serves as a favorable model for scientific research.

Although the precise mechanism of inflammasome activation is still elusive, there is no doubt that the consequences of inflammasome activation serve as an amplifier of neural inflammation in various aging‐related neurological diseases. Cleaved‐IL1β and cleaved‐IL18 released from inflammasome‐forming cells induce production of proinflammatory factors by neighbor cells, including IFNγ, TNFα, and ROS.^21^ The proinflammatory factors further impair blood‐brain barrier (BBB) and attract peripheral leukocytes.^22^, ^23^ Besides, pyroptosis induced by inflammasome activation gives rise to DAMPs, which activate inflammasome and exacerbate the inflammatory status. Neural inflammation plays a key role in multiple aging‐related neurological diseases. Accumulating evidence reveals that inflammasome activation could aggravate CNS disorders including neurodegenerative diseases and acute CNS stroke. Targeting inflammasome to dampen neural inflammation is a promising therapeutic strategy for the aging‐related neurological disorders.

## 3 MICROGLIA/MACROPHAGE ARE PRONE TO FORM INFLAMMASOME IN SENESCENT BRAINS IN THE ABSENCE OF DISEASE STATUS

Aging is accompanied by low‐grade inflammation in multiple organs, which is referred as “inflammaging”.^24^ The persistence of inflammatory status in aged CNS is related to chronic injury and the subsequent functional decline even in the absence of a specific disease. Recently, Furman et al^25^ reported that the status of inflammaging could be attributed to inflammasome activation. Inflammasome, the efficient inflammation amplifier, was inclined to get activated in aged microglia/macrophage. Multiple components of inflammasome, including NLRC4, Caspse‐1, Caspase‐11, ASC, and IL‐1β, were found to be increased in hippocampus of aged mice.^26^ In particular, expression of NLRP3, the most studied sensor of inflammasome, was increased in senile microglia compared with their young counterparts.^27^ Correspondingly, spontaneous activation of Caspase‐1 was detected in aged brain.^27^ The reason for inclination of inflammasome activation in aged microglia is related to the accumulation of a diverse array of endogenous metabolic danger signals in the microenvironment, including nucleotide metabolites (eg, such as adenine and N4‐acetylcytidine), uric acid, ATP, oxidative stress, cholesterol, lipotoxic fatty acids, and ceramides.^27^ Some mitochondrial microRNA (eg, let7b, mir‐146a, mir‐133b, mir‐106a, mir‐19b, mir‐20a, mir‐34a, mir‐181a, and mir‐221) and cellular microRNA (eg, miR‐146a, miR‐34a, and miR‐181a) are also involved in inflammasome‐dependent inflammaging.^28^

### 3.1 The consequences of inflammasome activation in aged microglia/macrophage are harsh

In aged mice without induction of any diseases, decrease of neurological functions was still evident due to accumulation of daily stress. Ablating NLRP3 inflammasome significantly improved cognitive and motor performance in IL‐1‐dependent manner.^27^ Recently, Furman et al^25^ found that the expression of specific inflammasome gene modules stratified the elderly into two extremes: those with high expression of inflammasome‐related genes and those without. The former population was found to have shorter longevity, high rate of hypertension, and arterial stiffness. The phenomenon that aged brains are prone to form inflammasome could be attributed to the development of neurodegenerative diseases and the violent exacerbation of acute neurological disorders in aged individuals. From the inflammasome, inclination in aged brains comes to the enlightenment that inhibiting chronic inflammasome activation might be a potent preventive and curative strategy for aging‐related CNS diseases. Since microglia/macrophage are the major source of inflammasome, targeting the particular cell type should be the most direct and efficient path for inflammasome suppression in aging‐related CNS disorders, in both chronic (eg, neurodegenerative diseases) and acute way (eg, CNS stroke).

## 4 INFLAMMASOME ACTIVATION IN MICROGLIA/MACROPHAGE IS THE RESULT OF NEURODEGENERATIVE DISEASE DEVELOPMENT AND PROMOTES THE DISEASE PROGRESSION

It is widely accepted that neurodegenerative diseases are associated with chronic CNS inflammation.^29^, ^30^ As an efficient inflammation amplifier, inflammasome activation could be the result of neurodegeneration as well as the reason for the disease progression. Specific components in neurodegenerative diseases, such as Amyloid β (Aβ) in Alzheimer's disease (AD) and α‐synuclein in Parkinson's disease (PD), promote inflammasome activation in microglia/macrophage. Meanwhile, activation of inflammasome results in production of highly proinflammatory cytokines such as IL‐1β and IL‐18 by microglia/macrophage, which accelerates disease development.

### 4.1 Inflammasome activation in Alzheimer's disease

AD causes memory loss and other cognitive impairment in the elderly. In the pathophysiological process of AD, inflammasome formation plays a negligible role. Inflammasome activation is the result of AD development. The extracellular plaque deposition of the Aβ is a principal event in the pathogenesis of AD. Deposition of Aβ causes lysosomal damage and ROS production in microglia, which induces inflammasome activation.^31^, ^32^, ^33^, ^34^ Moreover, it was reported that Aβ oligomers could directly interact with NLRP3 and ASC in infiltrated macrophages and cause inflammasome activation, which exacerbated neuroinflammation.^35^, ^36^ Increase of the inflammasome products (eg, IL‐1β) are detected in serum, cerebrospinal fluid, and brain tissue of AD patients.^37^ On the other hand, activation of inflammasome promotes AD development. For example, as a product of inflammasome, IL‐1β induces proinflammatory cytokines (eg, IL‐1β, IL‐6 and IL‐18) production and free radical release by glia cells, resulting in neurotoxicity.^38^ Meanwhile, IL‐1β promotes the production of β‐amyloid precursor protein (APP) and Aβ by neurons, induces phosphorylation of Tau protein, and mediates neurofibrillary tangle formation.^33^, ^39^, ^40^

After successive failure of clinical trial for new AD treatment, research on finding effective drug is imperative. Since inflammasome activation is associated with amyloid genesis and quantitative inflammation mediators, therapeutic approaches targeting inflammasome to postpone AD development have been proposed. Heneka et al^41^ found that interfering NLRP3 gene expression could reduce Aβ deposition, decrease neurotic plaque burden, and improve memory and behavior functions in AD models. Pharmacologically, suppressing inflammasome activation with ATP inhibitor or purinergic 2X7 (P_2_X_7_) receptor antagonist improved age‐related cognitive decline.^42^ The therapeutic effects of inflammasome suppressive treatments reveal that resisting inflammaging by tackling inflammasome is a promising strategy for AD therapy.

### 4.2 Inflammasome activation in Parkinson's disease

Parkinson's disease (PD) is a progressive degenerative CNS disorder that affects the motor system. It is characterized by a profound degeneration of dopaminergic neurons in the brain, accompanied by chronic neuroinflammation, mitochondrial dysfunction, and widespread accumulation of α‐synuclein‐rich protein in Lewy's bodies.^43^, ^44^, ^45^ It has been proved that the α‐synuclein is a potent stimulator of inflammasome. Fibrillar α‐synuclein in microglia leads to impairment of mitochondrial endocytosis and lysosomal dysfunction, which cause ROS dysregulation and activate inflammasome.^46^, ^47^ Besides, several kinds of microRNA (eg, miR‐7 and miR‐30e), which are increased in PD brains, could directly activate inflammasome in microglia.^46^, ^48^ Similar to the situation in AD, inflammasome products released by microglia within substantia nigra trigger the inflammatory cascades in PD brains. Processive inflammasome activation in microglia may be a sustained stimuli of neuroinflammation that drives progressive dopaminergic neuropathology and promotes fibrillar α‐synuclein accumulation.^49^, ^50^

Current therapies for PD, including levodopa treatment and deep brain stimulation, can only manage PD symptoms without tackling the pathological alterations in PD brains. The evidence that inflammasome plays a key role in pathophysiological process of PD highlights the potential of repressing inflammasome as a PD therapy.^51^ It has been proved that suppressing inflammasome activation with a treatment of NLRP3 inhibitor could effectively mitigate motor deficits, nigrostriatal dopaminergic degeneration, and α‐synuclein aggregation in PD model.^50^, ^52^ Inflammasome suppression may represent a thorough therapy that deals with the pathophysiology of PD.

## 5 INFLAMMASOME ACTIVATION INTENSIFIES NEURAL INFLAMMATION AFTER STROKE

Stroke, including ischemic and hemorrhagic subtypes, is one of the leading causes of death worldwide. As an aging‐related acute disorder, nearly three quarters of stroke occur in people over the age of 65.^53^ With aging, endothelial dysfunction occurs, followed by structural and functional alternation of cerebral microcirculation and microcirculation, increasing the risks for both ischemic and hemorrhagic stroke.^54^, ^55^ It has been demonstrated that phagocytic capacity is downregulated in senile microglia/macrophage. When hemorrhagic stroke occurs, the inflammatory milieu further limits phagocytic activities of microglia/macrophage, exacerbating disease severity of hemorrhagic stroke.^56^ Therefore, aging is an independent risk factor of both ischemic and hemorrhagic stroke and indicates detrimental disease outcomes.^57^ On the other hand, prognosis of stroke is largely dependent on the intensity of post‐stroke neural inflammation. Inflammasome that activates in CNS‐resident microglia and the infiltrated macrophage plays a decisive role in post‐stroke neural inflammation.

### 5.1 Inflammasome activation in acute ischemic stroke

**Figure 2:**
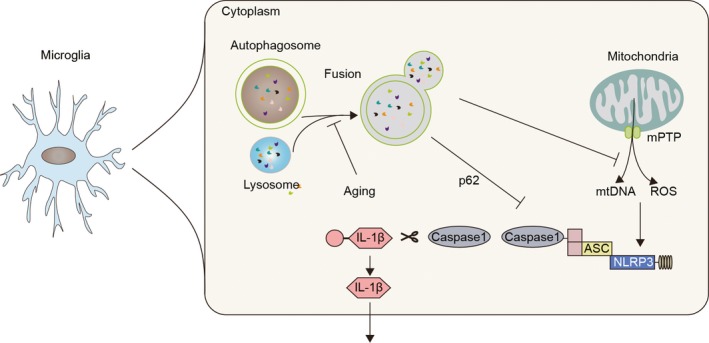
Inflammasome activation inhibits autophagy during aging. Autophagy can negatively regulate inflammasome activation by removing mitochondria‐derived stimuli. Aging impaired fusion of lysosomes with autophagosome, resulting in inflammasome activation by mitochondrial DNA (mtDNA) and ROS efflux from impaired mitochondria

**Figure 3:**
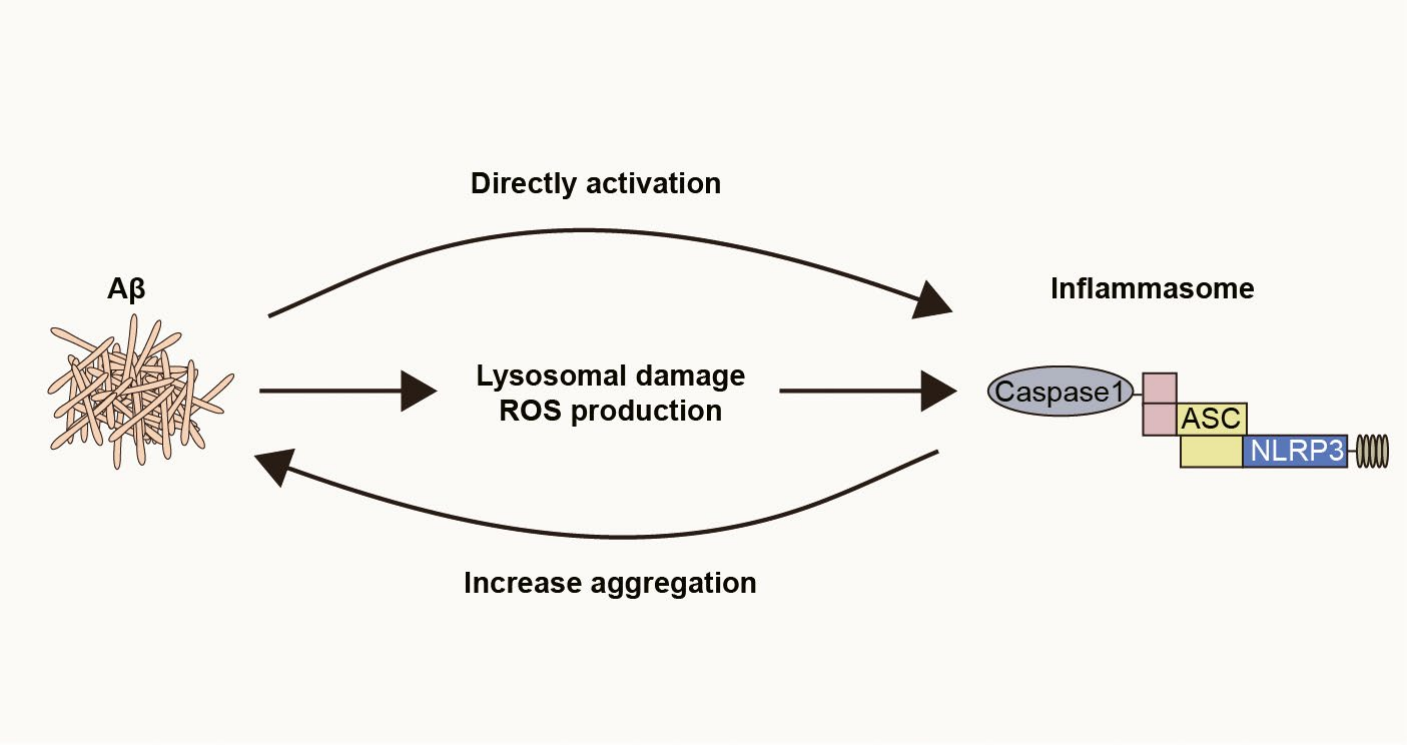
Vicious circle form between Aβ deposition and NLRP3 inflammasome activation in microglia/macrophage during AD. Inflammasome is activated in microglia/macrophage by Aβ directly, or by lysosomal damage and ROS production resulted from Aβ deposition. On the other hand, activation of inflammasome promotes Aβ aggregation exacerbating AD pathology

**Figure 4:**
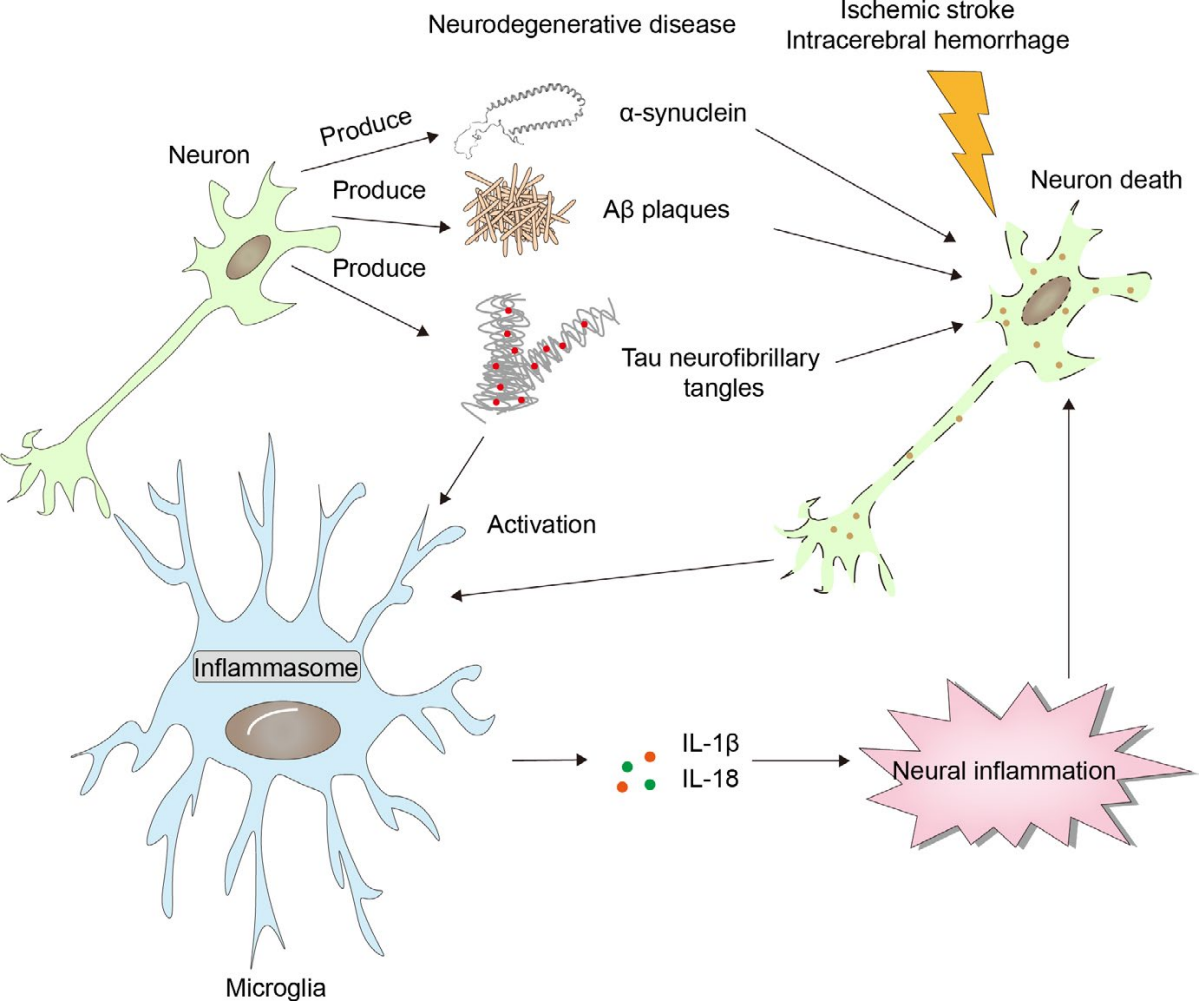
Interactions of inflammasome in microphage with neural inflammation and aging‐related diseases. Inflammasome activation in microglia/macrophage could be triggered by Aβ plague deposition and tau neurofibrillary tangle in AD, α‐synuclein in PD, and neurons necroptosis in CNS stroke. Therefore, inflammasome activation is one of the consequences of CNS disease development. On the other hand, inflammasome activation exacerbates to neural inflammation, which plays detrimental role in neurodegenerative diseases and CNS stroke. Thus, inflammasome activation is one of the reasons for CNS disease progression

Unlike the chronic neurodegenerative diseases, microglia/macrophage in stroke brain are confronted with robust pathological alterations. In ischemic stroke, acute deprivation of blood supply causes rapid necrosis of brain cells within the ischemic lesion. Rupture of necrotic brain cells gives rise to abundant DAMPs. The danger signals then directly activate inflammasome in microglia/macrophage through multiple pathways including NF‐kB and MAPK signaling.^58^, ^59^ Dysfunction of organelles during ischemic injury also contributes to inflammasome activation in microglia/macrophage. Dysfunction of the electron transport chain and accumulation of Ca^2+^ in the mitochondria result in robust ROS release. Stagnated ATP production then causes impairment of Na^+^/K^+^‐ATPase pumps which elicits potassium efflux in mitochondria. Rupture of lysosomal membrane leads to cathepsin leakage into the cytosol.^60^, ^61^, ^62^ ROS, potassium efflux, cathepsins, and DAMPs could all be sensed by inflammasome sensors and cause inflammasome activation. Physiologically, the process of autophagy serves as a natural extinguisher for inflammasome. With the autophagic adaptor of p62, autophagosome recruits inflammasome components and promotes their degradation in a lysosome‐dependent manner. In addition, autophagy removes damaged mitochondria through autophagosomes, preventing the release of ROS and mitochondrial DNA into the cytoplasm.^63^, ^64^, ^65^ In the lesion of ischemic stroke, the autophagic process is suppressed, which further facilitates inflammasome activation^65^ (Figures 2, 3 and 4). Activation of inflammasome in microglia/macrophage exerts detrimental impacts on brain tissue undergoing ischemic injury.^66^ As that in chronic neurogenerative diseases, inflammasome serves as an amplifier of post‐stroke neural inflammation. A cascade of inflammatory reactions is induced by inflammasome products released by microglia/macrophage, contributing to the BBB breakdown and the subsequent leukocyte infiltration.^67^

Accumulative evidences indicate that targeting inflammasome pharmacologically may salvage penumbral tissue in cerebral ischemia.^68^, ^69^ Remarkably, the NLRP3 inhibitor MCC950 and the NLRP1 neutralizing antibodies have been proved to be curative in ischemic stroke.^70^ Besides, repressing inflammasome through enhancing autophagy has also been proved to offer protection in ischemic stroke.^71^ Inhibiting inflammasome to limit excessive neural inflammation in acute ischemic stroke should be a promising therapeutic strategy, which needs further efforts for clinical translation.

### 5.2 Inflammasome and hemorrhagic transformation after ischemic stroke

Hemorrhagic transformation (HT) is one of the major complications of ischemic stroke. Activation of inflammasome during ischemic stroke promotes the occurrence of hemorrhagic transformation. Inflammasome products initial a robust inflammatory cascade, which exacerbates BBB injury, thus leading to vessel rupture and intracerebral bleeding.^72^ Currently, thrombolytic therapy is the only permissive pharmacological intervention for ischemic stroke. Nevertheless, patients that receive thrombolytic therapy have to confront increased risk of HT. Therefore, a strict time window is set up (4.5 hours) for thrombolytic treatment.^73^ Recently, it has been found that delayed recombinant tissue plasminogen activator (rtPA) treatment after ischemic stroke increased inflammasome activation in microglia thus increasing the risk of HT after ischemic stroke, which sheds light on the conclusion that inflammasome activation is the key limitation for therapeutic window for rtPA.^74^ Inhibiting inflammasome activation has displayed preventive efficacy for HT in ischemic stroke. NLRP3 inhibitor, MCC950, reduced the rate of HT in stroke animal models, accompanied by decreased leukocyte recruitment.^70^, ^75^ The evidence indicates that inflammasome inhibiting treatment may help to liberalize the time window for thrombolytic therapy through preventing HT occurrence.

### 5.3 Inflammasome activation in acute hemorrhagic stroke

Primary intracerebral hemorrhage (ICH) is another important subtype of acute stroke. Inflammasome plays a critical role in the pathophysiology of ICH‐induced brain injury. It has been found that inflammasome activates rapidly in microglia after the onset of ICH. As detected by Ma et al,^76^ inflammasome components, including NLRP3, cleaved Caspase‐1, and cleaved IL‐1β, were upregulated within 3 hours after ICH. As products of blood degradation, iron and heme initiate inflammasome activation in ROS‐dependent manner.^77^ Mitochondrial dysfunction is proved to be the major trigger for inflammasome activation in microglia during ICH pathology.^76^ Besides, miR‐223 has been demonstrated to directly regulate NLRP3 expression in ICH.^78^ As that in acute ischemic stroke, inflammasome products exacerbate BBB injury and promote leukocyte recruitment, thus enlarging perihematomal edema and exacerbating brain injury.^76^, ^79^ Targeting NLRP3 inflammasome activation may be a promising therapeutic strategy for ICH.^80^, ^81^ The P_2_X_7_ receptor is upstream of NLRP3 activation, and its inhibition has a pronounced neuroprotective effect in an ICH rat model.^80^ Blocking Caspase‐1 signaling or NLRP3 inflammasome has been demonstrated to effectively reduce the inflammatory responses and improved disease outcomes in ICH.^82^, ^83^

Phagocytosis of hematoma by microglia/macrophage is important to remove hematoma and ameliorate neuroinflammation after ICH. It is reported that phagocytosis, with abundant ROS production, could activate NLRP3 inflammasome in microglia/macrophage.^84^, ^85^ However, the impact of inflammasome activation on microglia/macrophage phagocytic activities remains elusive. Inflammasome activation in microglia/macrophage represents a certain kind of proinflammatory activity. It is well recorded that the proinflammatory phenotype of microglia/macrophage displays downregulated phagocytic capacity. On the other hand, it is the inflammation‐resolving microglia/macrophage that bears the full capacity of phagocytosis. Therefore, we infer that inhibiting inflammasome activation could protect microglia/macrophage from the self‐injury during phagocytosis and keep microglia/macrophage in the inflammatory‐resolving phenotype which facilitates hematoma clearance.

## 6 TARGETING INFLAMMASOME TO RETARD THE PACE OF AGING AND BRAKE AGING‐RELATED CNS DISEASES

Aging and the associated disorders prime CNS‐resident microglia and infiltrated macrophage for inflammasome activation during “healthy” and disease status. Conversely, inflammasome accelerates the aging process and exacerbates aging‐associated CNS diseases. Metabolism disorder is frequently observed in the elderly. As mentioned, accumulation of multiple metabolites could trigger inflammasome activation.^25^, ^27^ The other way round, activation of inflammasome exacerbates the status of metabolic disorder. Inflammasome contributes to glucose homeostasis, drives catecholamine degradation, blunts lipolysis, which causes visceral adiposity, and decreases substrate mobilization in the aged individuals.^86^ Therefore, developing lifestyle could prevent inflammasome activation, thus retards the pace of aging. It is evident that increased caffeine intake is relevant to lower inflammasome activation.^25^ Caloric restriction or a low‐carbohydrate ketogenic diet could inhibit inflammasome activation through elevating the level of β‐hydroxybutyrate.^87^ Proper physical exercise would prevent the induction of inflammasome.^88^ Sound sleep also helps to keep inflammasome quiescent.^89^ Confronting the aging‐related CNS diseases, targeting inflammasome is a promising therapeutic strategy. A broad body of evidences suggest that inhibiting inflammasome activation displays favorable curative effects in neurodegenerative diseases 42, 50, 52 as well as acute CNS injuries.^70^, ^75^, ^82^, ^83^

## 7 CONCLUSION

Senescence and the concomitant “inflammaging” are both the reason and the result of CNS inflammasome activation. CNS‐resident microglia and peripheral macrophage are the main cell types in which inflammasome activation is initiated. In “healthy” aged brains and CNS with aging‐related diseases, inflammasome activation in microglia/macrophage is broadly observed. Preventing inflammasome activation by improving lifestyle should be favorable for delaying senility. Inhibiting inflammasome activation with pharmacological interventions may help put on the brake for aging‐related CNS diseases.

## CONFLICT OF INTEREST

The authors declare no conflicts of interest.

## AUTHOR CONTRIBUTION

MH and YL wrote the manuscript. BZ and DL conducted the manuscript editing. WC and ZL designed and critically revised the manuscript. All authors read and approved the final manuscript.
